# Evaluation of Suicide Mortality Among Sexual Minority US Veterans From 2000 to 2017

**DOI:** 10.1001/jamanetworkopen.2020.31357

**Published:** 2020-12-28

**Authors:** Kristine E. Lynch, Elise Gatsby, Benjamin Viernes, Karen C. Schliep, Brian W. Whitcomb, Patrick R. Alba, Scott L. DuVall, John R. Blosnich

**Affiliations:** 1 Veterans Affairs (VA) Informatics and Computing Infrastructure, VA Salt Lake City Health Care System, Salt Lake City, Utah; 2 Division of Epidemiology, Department of Internal Medicine, The University of Utah, Salt Lake City; 3 Department of Family and Preventive Medicine, The University of Utah, Salt Lake City; 4 Department of Public Health and Health Sciences, University of Massachusetts, Amherst; 5 Suzanne Dworak-Peck School of Social Work, University of Southern California, Los Angeles; 6 Center for Health Equity Research and Promotion, VA Pittsburgh Healthcare System, Pittsburgh, Pennsylvania

## Abstract

**Importance:**

Identification of subgroups at greatest risk for suicide mortality is essential for prevention efforts and targeting interventions. Sexual minority individuals may have an increased risk for suicide compared with heterosexual individuals, but a lack of sufficiently powered studies with rigorous methods for determining sexual orientation has limited the knowledge on this potential health disparity.

**Objective:**

To investigate suicide mortality among sexual minority veterans using Veterans Health Administration (VHA) electronic health record data.

**Design, Setting, and Participants:**

This retrospective population-based cohort study used data on 8.1 million US veterans enrolled in the VHA after fiscal year 1999 that were obtained from VHA electronic health records from October 1, 1999 to September 30, 2017. Data analysis was carried out from March 1, 2020 to October 31, 2020.

**Exposure:**

Veterans with documentation of a minority sexual orientation. Documentation of sexual minority status was obtained through natural language processing of clinical notes and extraction of structured administrative data for sexual orientation in VHA electronic health records.

**Main Outcomes and Measures:**

Suicide mortality rate using data on the underlying cause of death obtained from the National Death Index. Crude and age-adjusted mortality rates were calculated for all-cause death and death from suicide among sexual minority veterans compared with the general US population and the general population of veterans.

**Results:**

Among the 96 893 veterans with at least 1 sexual minority documentation in the electronic health record, the mean (SD) age was 46 (16) years, 68% were male, and 70% were White. Of the 12 591 total deaths, 3.5% were from suicide. Veterans had a significantly higher rate of mortality from suicide (standardized mortality ratio, 4.50; 95% CI, 4.13-4.99) compared with the general US population. Suicide was the fifth leading cause of death in 2017 among sexual minority veterans (3.8% of deaths) and the tenth leading cause of death in the general US population (1.7% of deaths). The crude suicide rate among sexual minority veterans (82.5 per 100 000 person-years) was higher than the rate in the general veteran population (37.7 per 100 000 person-years).

**Conclusions and Relevance:**

The results of this population-based cohort study suggest that sexual minority veterans have a greater risk for suicide than the general US population and the general veteran population. Further research is needed to determine whether and how suicide prevention efforts reach sexual minority veterans.

## Key Points

**Question:**

How does suicide mortality among sexual minority veterans compare with suicide mortality in the general US and veteran populations?

**Findings:**

In this cohort study of 96 893 sexual minority veterans, risk of death from suicide was higher among sexual minority veterans compared with the general US population and the general veteran population.

**Meaning:**

These findings suggest that sexual minority veterans have an increased risk of suicide mortality and that understanding whether and to what extent prevention efforts reach this population should be the focus of future research.

## Teaser

This cohort study compares suicide mortality among sexual minority veterans with suicide mortality in the general US and veteran populations.

## Introduction

Suicide is a widespread public health concern and remains a priority for the Veterans Health Administration (VHA). In 2017, the suicide rate among veterans was 1.5 times higher than that in the nonveteran US population.^1^ Like any population, the veteran community is heterogeneous, comprising various subgroups with unique health needs and experiences, and suicide disproportionally affects certain groups of veterans. For example, suicide rates are higher among male veterans than among female veterans, and the suicide rates among those with psychiatric diagnoses are more than 2 times higher than the rate in the general veteran population.^2^ Among transgender veterans, a historically stigmatized population, not only is the suicide rate higher than among cisgender veterans, on average, suicide occurs at younger ages.^3^ People with gay, lesbian, or bisexual sexual orientation (ie, sexual minority [SM] individuals) also have a higher lifetime prevalence of suicide attempts, and limited research suggests that they have a greater risk for death by suicide.^4,5,6^

Sexual minority individuals have a higher lifetime risk for suicide ideation and attempts than heterosexual individuals,^7,8,9,10,11^ but suicide data for SM populations are not well characterized. Lack of reliable mortality data has been an obstacle to understanding mortality disparities experienced by SM communities, including SM veterans. In addition to the lack of inclusion of sexual orientation in mortality surveillance data,^12^ data including designation of sexual orientation at the population level are limited. In previous work,^13^ despite the VHA’s current lack of systematic collection methods for sexual orientation, we found patient sexual orientation documented in clinical notes and, to a lesser extent, with administrative coding (eg, *International Statistical Classification of Diseases and Related Health Problems, Tenth Revision [ICD-10]* codes), culminating in a cohort of more than 100 000 SM veterans.

Sexual minority veterans likely have the same risk factors for suicide as non-SM veterans (eg, posttraumatic stress disorder^14^), but they also contend with historical institutional stigma^15^ that may influence mortality by suicide, a framework known as minority stress. Minority stress posits that SM populations experience poorer health than heterosexual populations because of distress associated with societal and interpersonal discrimination, prejudice, and violence.^16^ The compounding effects of minority stress may contribute to excess death by suicide among SM veterans; however, to our knowledge, no studies have examined suicide mortality among veterans based on SM status.

Drawing from previous work,^13^ we assessed suicide rates among a cohort of veterans with SM sexual orientation documented within the VHA electronic health record (EHR). Based on previous research suggesting that suicidal ideation and suicide attempts are more frequent among SM individuals than among heterosexual individuals^10^ and that previous suicide attempt is 1 of the strongest correlates of death from suicide,^17^ we hypothesized that SM veterans would have a higher rate of suicide than the general US and veteran populations.

## Methods

This cohort study used data sourced from an existing retrospective cohort of SM veterans who use VHA for health care^13^ that were obtained from the VHA Corporate Data Warehouse, a data repository with historical nationwide data beginning October 1, 1999.^18^ This study was approved by the University of Utah institutional review board. Because the study was retrospective and posed no more than minimal risk to participants, the requirement for informed consent was waived. The study followed the Strengthening the Reporting of Observational Studies in Epidemiology (STROBE) guideline.^19^

Veterans who enrolled in the VHA after fiscal year (FY) 1999 who had a record of at least 1 outpatient VHA encounter and documentation of SM sexual orientation in FY2000 to FY2017 were considered for analysis. Documentation was defined by record of SM sexual orientation according to administrative data (*International Classification of Diseases, Ninth Revision* codes: 302.0 and 302.52; *ICD-10* codes: Z72.52 and Z72.53) and clinical notes extracted via natural language processing. The specifications of the natural language processing extraction process are described elsewhere.^13,20^ Concept dictionaries were used to identify all known terms used to describe sexual orientation. A natural language processing system composed of rule-based and machine learning classifiers was used to identify and categorize instances of the terms. The output contained instance-level categorizations of SM documentation, which were summarized to the patient level for the present study.

Patients with at least 1 instance of SM sexual orientation documentation were considered SM veterans, and the index date was defined as the date of first documentation either by code or note. Structured birth sex data from the VHA Corporate Data Warehouse were used to categorize veterans as SM men or SM women. To approximate a comparison group for subanalysis, veterans without SM sexual orientation documentation were matched 1:1 without replacement to the SM cohort in the FY of the index date (based on a random inpatient or outpatient visit date for the former group), age, and sex.

National Death Index (NDI) data were only available for SM veterans and were used to obtain vital status and primary cause and date of death for SM veterans. Primary cause of death was based on *ICD-10* codes according to the underlying cause of death reported by the NDI and evaluated according to code groupings.^21^ Deaths by suicide were defined by *ICD-10* codes X60-84 and Y87.0. Person-years accrued from the index date until death or September 30, 2017 (end of observation period).

### Statistical Analysis

Crude all-cause and suicide mortality rates were calculated overall, by FY, and by patient sex. Age-adjusted rates were calculated with direct standardization per US 2000 Census Bureau population data.^22^

For the comparative subanalysis, dates of death for the matched sample of veterans without SM documentation were obtained from the VHA Vital Status File. The Vital Status File does not include data about cause of death; thus, comparisons could only be done for all-cause mortality. Cox proportional hazards regression analyses were used to compare all-cause survival distributions between SM veterans and veterans without SM documentation.

Suicide mortality among SM veterans was compared with previously published data on mortality in the US population and the general VHA population.^2^ For comparison of rates with that in the US population, rank order of cause-specific leading causes of death among SM veterans was compared with that in the general US population in 2017 as reported by the National Center for Health Statistics.^21^ For the top 10 causes of death among SM veterans, age-adjusted standardized mortality ratios and 95% CIs were obtained by multiplying the total person-years accrued in the SM cohort by US 2017 cause-specific mortality rates^23^ and summed across age groups (18-34 years, 35-64 years, and >64 years). To isolate differences in suicide mortality that might be attributed to sexual orientation, a subanalysis was performed to compare suicide rates found among SM veterans with previously reported rates among all VHA veterans (37.7 per 100 000 person-years).^2^ The previously published study, which also used data from the VHA EHR, was a closed cohort study of all veterans who used VHA in FY1999 and were alive at the start of FY2000. Patients were followed up for up to 7 years. For the present analysis, to generate a consistent secular comparison, the SM cohort was restricted to patients with a health care visit in the first year of available data, FY2000, and alive at the start of FY2001. Person-years accrued from the start of FY2001 until time of death or the end of FY2007, whichever came first. Overall crude suicide rates were calculated per 100 000 person-years. All analyses were performed using R, version 3.6.1 (R Project for Statistical Computing). The *P* values were 2 sided, with statistical significance set at *P* < .05. Data analysis was performed from March 1, 2020 to October 31, 2020.

## Results

Of the 8.2 million veterans assessed, 96 893 veterans (68% men; 70% White; mean [SD] age, 46 [16] years) had at least 1 documentation of SM status from FY2000 to FY2017. Sexual minority women veterans were significantly younger on the index date compared with SM men veterans (39.3 vs 50.3 years), more likely to be Black (26.1% vs 16.8%), and more likely to be alive at the end of FY2017 (96.1% vs 82.9%) (Table 1). Among the decedents, SM women were younger than SM men (mean [SD] age at death, 59.1 [16.59] years vs 67.4 [14.25] years).

**Table 1.** Characteristics of Sexual Minority Veterans From Fiscal Years 2000 to 2017^a^

Characteristic	All sexual minority persons (N = 96 893)	Sexual minority women (n = 30 655)	Sexual minority men (n = 66 238)
Sex			
Male	66 238 (68.4)	0 (0.0)	66 238 (100.0)
Female	30 655 (31.6)	30 655 (100.0)	0 (0.0)
Race			
American Indian or Alaskan native	890 (0.9)	364 (1.2)	526 (0.8)
Asian	1031 (1.1)	396 (1.3)	635 (1.0)
Black	19 143 (19.8)	7993 (26.1)	11 150 (16.8)
Native Hawaiian or other Pacific Islander	861 (0.9)	327 (1.1)	534 (0.8)
Unknown	7065 (7.3)	2243 (7.3)	4822 (7.3)
White	67 903 (70.1)	19 332 (63.1)	48 571 (73.3)
Ethnicity			
Hispanic	8771 (9.1)	3130 (10.2)	5641 (8.5)
Not Hispanic	83 505 (86.2)	26 279 (85.7)	57 226 (86.4)
Unknown	4617 (4.8)	1246 (4.1)	3371 (5.1)
Age at index sexual minority documentation, y			
Mean (SD)	46.79 (16.10)	39.31 (12.86)	50.26 (16.27)
18-29	17 857 (18.4)	8905 (29.0)	8952 (13.5)
30-39	18 652 (19.3)	8216 (26.8)	10 436 (15.8)
40-49	17 673 (18.2)	6272 (20.5)	11 401 (17.2)
50-59	20 356 (21.0)	5150 (16.8)	15 206 (23.0)
60-69	14 345 (14.8)	1653 (5.4)	12 692 (19.2)
70-79	5091 (5.3)	270 (0.9)	4821 (7.3)
≥80	2919 (3.0)	189 (0.6)	2730 (4.1)
Dead or alive			
Alive on September 30, 2017	84 302 (87.0)	29 454 (96.1)	54 848 (82.8)
Dead	12 591 (13.0)	1201 (3.9)	11 390 (17.2)
Age at death, y			
Mean (SD)	66.64 (14.69)	59.13 (16.59)	67.43 (14.25)
18-29	146 (1.2)	43 (3.6)	103 (0.9)
30-39	430 (3.4)	101 (8.4)	329 (2.9)
40-49	830 (6.6)	144 (12.0)	686 (6.0)
50-59	2429 (19.3)	396 (33.0)	2033 (17.8)
60-69	3557 (28.3)	256 (21.3)	3301 (29.0)
70-79	2373 (18.8)	79 (6.6)	2294 (20.1)
≥80	2826 (22.4)	182 (15.2)	2644 (23.2)

^a^ Includes only SM veterans with documentation of sexual minority sexual orientation in the Veterans Health Administration electronic medical record.

From FY2000 to 2017, 12 591 SM veterans (13.0%) died. Sexual minority men had significantly higher crude mortality than SM women (3077.40 [95% CI, 3021.14-3134.45] per 100 000 person-years] vs 757.70 [95% CI, 715.45-801.80] per 100 000 person-years) (Table 2). By the end of FY2017, 436 SM veterans had died by suicide (unadjusted mortality: 82.47 [95% CI, 74.92-90.60] per 100 000 person-years; age-adjusted mortality: 74.73 [95% CI, 67.15-82.93] per 100 000 person-years), with SM veteran men having a higher rate than SM veteran women: 346 men died by suicide (unadjusted mortality: 93.48 [95% CI, 83.89-103.87] per 100 000 person-years; age-adjusted mortality: 100.14 [95% CI, 89.14-112.21] per 100 000 person-years) compared with 90 women (unadjusted mortality: 56.78 [95% CI, 45.66-69.79] per 100 000 person-years; age-adjusted mortality: 49.32 [95% CI, 39.23-61.21] per 100 000 person-years) (Table 2). Both crude and age-adjusted suicide rates fluctuated throughout the 18-year period. Suicide rates increased from 60.86 per 100 000 person-years (95% CI, 40.76-87.42) in 2013 to 81.72 per 100 000 person-year (95% CI, 63.07-104.17) in 2017. The Figure presents crude and age-standardized suicide rates. Of the 96 893 matched veterans without SM documentation, 8689 (8.9%) died. The median follow-up time was 4.8 years (range, 2.00-8.59), and SM veterans had significantly higher mortality risk compared with veterans without SM documentation (hazard ratio, 1.59; 95% CI, 1.55-1.64; *P* < .001).

**Table 2.** Crude and Age-Adjusted Mortality Rates Overall and by Sex Among Sexual Minority Veterans^a^

Sex	All deaths, No.	Total person-years	All-cause mortality rate, per 100 000 person-years (95% CI)	Deaths by suicide, No.	Suicide mortality rate, per 100 000 person-years (95% CI)
Crude					
All	12 591	528 623.21	2381.85 (2340.42-2423.82)	436	82.47 (74.92-90.60)
Men	11 390	370 117.20	3077.40 (3021.14-3134.45)	346	93.48 (83.89-103.87)
Women	1201	158 506.02	757.70 (715.45-801.80)	90	56.78 (45.66-69.79)
Age-adjusted					
All	12 591	528 623.21	2699.05 (2603.81-2796.89)	436	74.73 (67.15-82.93)
Men	11 390	370 117.20	3443.61 (3370.96-3517.44)	346	100.14 (89.14-112.21)
Women	1201	158 506.02	1954.49 (17809.75-2140.61)	90	49.32 (39.23-61.21)

^a^ Direct standardization per US 2000 Census. Includes only sexual minority veterans with documentation of sexual minority sexual orientation in Veterans Health Administration electronic health records.

**Figure.**
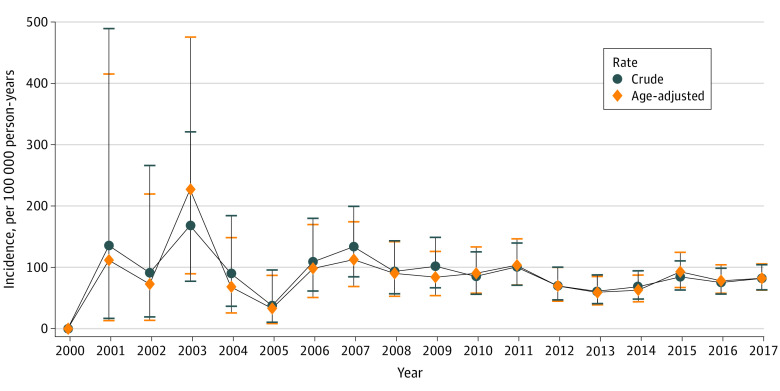
Crude and Age-Adjusted Suicide Rates Among Sexual Minority Veterans From Fiscal Years 2000 to 2017 Error bars indicate 95% CIs.

The ranked causes of death differed between SM veterans and the general US population in 2017 (Table 3). In 2017, suicide accounted for 3.8% of deaths (fifth most common cause of death) in the SM veteran population compared with 1.7% in the general US population (tenth most common cause of death). There was also excess mortality across every cause of death category among SM veterans compared with the US population. Calculated standardized mortality ratios ranged from 2.27 (95% CI, 1.93-2.66) for kidney disease to 5.55 (95% CI, 4.96-6.19) for Alzheimer disease, with the standardized mortality ratio death by suicide being higher (4.50; 95% CI, 4.13-4.99) than would be expected given the age-specific rates in the US population (Table 3).

**Table 3.** Mortality Among SM Veterans by Causes of Death Compared With the General US Population

*ICD-10* code	Cause of death	SM veterans in 2000-2017 (n = 12 591)^a^		General US population in 2017^b^		SM veterans in 2017 (n = 1276)		SM veteran deaths in 2000-2017		
		%	Rank	%	Rank	%	Rank	No.		SMR (95% CI)
								Observed	Expected^c^	
C00-C97	Malignant neoplasms (cancer)	22.0	1	21.3	2	21.1	2	2770	826.65	3.35 (3.32-3.47)
I00-I09, I11, I13, and I20-I51	Diseases of the heart	19.5	2	23.0	1	21.8	1	2457	648.77	3.78 (3.64-3.93)
V01-X59 and Y85-Y86	Accidents (unintentional injuries)	6.9	3	6.0	3	8.2	3	873	330.91	2.82 (2.64-3.02)
J40-J47	Chronic lower respiratory tract diseases	5.2	4	5.7	4	4.9	4	648	202.39	3.20 (2.96-3.45)
I60-I69	Cerebrovascular diseases (stroke)	3.6	5	5.2	5	2.9	7	456	182.36	2.50 (2.27-2.73)
U03, X60-X84, and Y87.0	Intentional self-harm (suicide)	3.5	6	1.7	10	3.8	5	436	95.81	4.50 (4.13-4.99)
E10-E14	Diabetes mellitus	3.3	7	3.0	7	3.6	6	420	124.39	3.37 (3.06-3.71)
G30	Alzheimer disease	2.5	8	4.3	6	2.3	8	313	56.38	5.55 (4.96-6.19)
J09-J18	Influenza and pneumonia	1.6	9	2.0	8	1.7	9	200	69.81	2.86 (2.48-3.28)
N00-N07, N17-N19, and N25-N27	Kidney disease	1.2	10	1.8	9	1	10	150	65.93	2.27 (1.93-2.66)
All other codes	All other causes	30.7	NA	26.0	NA	28.7	NA	NA	NA	NA

Abbreviations: *ICD-10*, *International Statistical Classification of Diseases and Related Health Problems, Tenth Revision (ICD-10)*; SM, sexual minority; SMR, standardized mortality ratio; NA, not applicable.

^a^ Includes only SM veterans with documentation of SM sexual orientation in Veterans Health Administration electronic health records.

^b^ Source: National Center for Health Statistics, National Vital Statistics, Mortality.^21^

^c^ Expected death counts were calculated using the 2017 WONDER, Centers for Disease Control and Prevention rates with adjustment for age.^23^

The mean (SD) age at suicide was 46.85 (14.15) years, with SM women being younger at the time of death by suicide (mean [SD] age, 42.0 [12.61] years) than SM men (mean [SD] age, 47.9 [4.33] years). Although suicide was the sixth most common cause of death among all SM veterans from 2000 to 2017, for both SM men and SM women, suicide was the most common cause of death among those 18 to 29 years of age, accounting for 39% of all deaths in the age group. The most common method of suicide was use of a firearm (39.6%), with men more likely than women (41.0% vs 34.5%) to use firearms but women more likely than men to die by poisoning (42.2% vs 26.4%) (Table 4).

**Table 4.** Method of Suicide Among Sexual Minority Veterans From Fiscal Years 2000 to 2017^a^

Method	Sexual minority persons, No. (%)		
	All	Men	Women
Firearm	174 (39.6)	143 (41.3)	31 (34.4)
Poisoning	130 (29.6)	92 (26.6)	38 (42.2)
Suffocation	91 (20.7)	72 (20.8)	19 (21.1)
Other	41 (10.0)	39 (11.3)	2 (2.2)
Total	436 (100)	346 (100)	90 (100)

^a^ Includes only sexual minority veterans with documentation of sexual minority sexual orientation in Veterans Health Administration electronic health records.

In the closed cohort subanalysis, 634 SM veterans were active patients in the VHA in FY2000 and alive at the start of FY2001. Of those patients, 4 died by suicide in the 7-year period, with an overall suicide rate of 102.96 per 100 000 person-years, more than 2 times higher than what was reported in the general VHA population (37.7 per 1000 person-years).^2^

## Discussion

Innovative use of EHR data can fill gaps in knowledge about SM health caused by the exclusion of sexual orientation from mortality surveillance.^24^ The VHA is an ideal national laboratory given its breadth and ability to use NDI data. Among SM veterans with documentation of sexual orientation, estimates of suicide mortality were more than 2 times higher than those in the general VHA population.^2^ Although not the primary focus of this study, increases in all causes of death were also observed. The large disparities suggest that SM veterans should be a priority population for prevention efforts.

Evaluating the extent to which deaths by suicide are decreasing and suicide prevention efforts are improving is dependent on consistent surveillance efforts. Although literature on suicide among SM populations is scant, the present results corroborate previous limited research on suicide mortality. For example, studies of national partnership registries from Sweden and Denmark have revealed greater risks of suicide among SM individuals (defined as adults with same-sex partners) compared with heterosexual individuals (persons with opposite-sex partners).^4,5^ Research in the US using survey data paired with mortality data has revealed greater risk of suicide among SM women compared with heterosexual women.^6^ However, not all studies have detected statistically significant differences in suicide between SM and heterosexual individuals.^25^ Although some discordance may be attributable to small sample sizes, the period of observation, or operationalization of SM status, the overall summary is seemingly unequivocal. Additional clarity hinges on more research, especially with adequate samples of SM individuals, appropriate comparison groups, and structural improvements in mortality surveillance to include sexual orientation.^12^ Although EHR data provide an opportunity to evaluate SM health outcomes, the best way to identify an appropriate comparison group is not clear. Veterans without documentation of SM status were included in the present analysis as an internal comparison group for all-cause mortality, but exploration of the impact of varying comparator definitions using EHR data (eg, those without SM documentation, those with explicit heterosexual documentation) should be a focus of future work.

Suicide rates among SM veterans align with rates found among transgender veterans who used VHA services between 2000 and 2009 (82 per 100 000 person-years), as does age at suicide (46 years of age in both studies).^3^ The VHA continues to enhance the quality and delivery of care offered to transgender and SM veterans, and both populations are increasingly seeking care at the VHA,^26^ but it is currently unknown how SM veterans reach VHA suicide prevention resources and how suicide prevention resources are provided to them. The VHA has mobilized an infrastructure around suicide prevention, including the Veterans Crisis Line, a network of suicide prevention coordinators at all VHA medical centers and large community-based outpatient clinics, and a data surveillance program to monitor suicidal ideation and attempts among veterans receiving VHA care.^27,28^ However, because information on sexual orientation is not collected like other demographic data, understanding the reach, access, and use of these suicide prevention resources by SM veterans is hampered.

These results raise important issues for future research. For example, because SM women are overrepresented in military and veteran populations, more in-depth study could explore whether and how differences in age at the time of death by suicide may be affected by the changing demographics of these populations over time. In addition, racial/ethnic differences could not be explored because of the sparseness of data, but additional years of data coupled with increasing SM documentation could ultimately provide data sets large enough to power intersectional analyses.

Sexual minority populations experience stressors associated with increased risk for suicide, including higher rates of depression and adverse social determinants of health that are often associated with suicide risk, such as adverse childhood experiences, adulthood sexual violence, and homelessness.^29,30^ The death disparities presented in this study suggest a need for future research to identify key modifiable risk factors for suicide among SM veterans. Depression seems to be the most likely candidate because it can be identified in medical record data. Conversely, identifying socially based risk factors for suicide in EHR data is challenging, although recent research suggests potential.^31,32,33^ Incorporating standardized data elements about social determinants of health, as suggested by the National Academy of Medicine,^34^ could propel health services–based suicide prevention and may be particularly helpful for health disparities research.

Characterizing populations according to method of suicide is advantageous for prevention efforts and for identifying those who stand to benefit most from targeted efforts.^35^ The number of deaths by suicides among SM veterans due to poisoning and suffocation was higher than that in the general VHA population.^1^ The most common method of suicide among all veterans who died by suicide in 2017 was self-inflicted firearm injury, accounting for nearly 70% of deaths by suicide among men and 43% among women. Although suicide by firearm was the most common method for SM men, the prevalence was only 40%. Among female SM veterans, death by poisoning was the most common cause of suicide, with a mortality almost 2 times higher than what was recently reported among a nationally representative sample of lesbian decedents.^36^

Under ideal circumstances, sexual orientation data would be collected no differently than other sociodemographic characteristics, especially given the research documenting numerous health disparities affecting SM populations.^37^ Several best-practice guides and cognitive interview studies for gathering sexual orientation data exist,^38^ including those specific to health care systems.^39^ Moreover, previous research suggests that veterans are no more or less likely than nonveterans to refuse to answer sexual orientation questions in surveys.^40^ The VHA should investigate methods to routinely collect sexual orientation information in its administrative data to better serve SM veterans.

Currently, no evidence-based suicide prevention programs specific to SM populations exist.^41^ Research is needed to determine how suicide prevention efforts reach SM veterans and whether tailored approaches may be needed. Standardized data elements in the EHR enabling veterans to self-report sexual orientation and fostering affirming clinical environments in which they can do so will be critical to facilitate intervention and research to address disparities in suicide.

### Limitations

This study has limitations. The present study used EHR data to identify SM status and NDI data to define suicide mortality; both have inherent limitations that may affect study findings and warrant discussion. First, this study included only SM veterans who have used VHA services and had documentation of SM sexual orientation in their health records. Although our approach to extract instances of sexual orientation was rigorously developed and validated,^20^ the ability of the natural language processing system to adequately extract SM status depends on multiple components, including the existence of the information in the notes. In other words, SM veterans without SM status documentation in their EHRs were not evaluated. Before the repeal of Don’t Ask Don’t Tell in September 2011, revealing homosexual behavior to a military health care provider could have had repercussions, including dishonorable discharge.^42^ In the records of the thousands of discharges that occurred,^29^ service members conveyed how a heightened culture of fear and secrecy emerged around them from the suspicion of SM status because of the use of SM allegations to punish service persons.^43,44,45^ Although Don’t Ask Don’t Tell did not apply to the VHA, residual effects have been documented among veterans, including reluctance to disclose sexual orientation information to VHA providers.^46^ Because factors associated with SM nondisclosure, such as decreased psychological well-being,^47^ are also associated with suicide risk, the results may underestimate suicide risk among all SM veterans. Second, although this study includes examination of 1 of the largest nationwide cohorts of SM veterans to date, the variability in sexual orientation documentation precluded more granular assessment of SM status. Sexual orientation has multiple dimensions including identification, behavior, and attraction,^48^ and heterogeneity across these dimensions could not be evaluated in the present study. Third, suicide mortality was identified by cause of death data according to *ICD-10* coding extracted from death certificates, which may be inexact. For some causes, such as coronary heart disease and cardiovascular disease, NDI data overestimate the number of deaths attributed to these causes compared with expert adjudication.^49^ With suicide, conversely, a recent study of death certificate data in Utah revealed that 33% of deaths classified as deaths due to overdose were misclassified and were actually deaths by suicide.^50^ Whether the extent of misclassification observed in Utah extends to other states is unclear, but some degree of under-ascertainment of suicides in this study is possible.

## Conclusions

The results of this cohort study suggest that sexual minority veterans have a greater risk for suicide than both the general US population and the general veteran population. Further research is needed to determine whether and how suicide prevention efforts reach sexual minority veterans.
